# Impact of a Cystic Fibrosis Specific Multivitamin Formulation on Fat-Soluble Vitamin Status and Treatment Satisfaction in Young Children

**DOI:** 10.3390/children12091149

**Published:** 2025-08-29

**Authors:** Anne Munck, Raphael Enaud, Jeanne Languepin, Natascha Remus, Nathalie Wizla, Frederique Chedevergne, Stephanie Bui, Amelie Arrouy, Marie Mittaine, Megan Quinn, Amy Wahlquist, Isabelle Sermet-Gaudelus

**Affiliations:** 1 Hospital Necker Enfants Malades, CF Center, 75015 Paris, France; 2 Hospital Pellegrin-Enfants, CF Center, CIC, 33000 Bordeaux, France; raphael.enaud@chu-bordeaux.fr; 3 CHU Dupuytren, CF Center, 87000 Limoges, France; jeanne.languepin@chu-limoges.fr; 4 Intercommunal Hospital, Center on Rare Respiratory Diseases, 94000 Creteil, France; natacha.remus@chicreteil.fr; 5 University Hospital Lille, Paediatric CF Center, 59037 Lille, France; nathalie.derambure@chru-lille.fr; 6 Hopital Necker-Enfants Malades, CF Center, 75015 Paris, France; frederique.chedevergne@aphp.fr; 7 Hopital Pellegrin, Paediatric CF Center, 33000 Bordeaux, France; stephanie.bui@chu-bordeaux.fr; 8 University Hopital des Enfants, CIC, 31300 Toulouse, France; arrouy.a@chu-toulouse.fr; 9 University Hopital des Enfants, Paediatric CF Center, 31300 Toulouse, France; mittaine.m@chu-toulouse.fr; 10 Department of Biostatistics and Epidemiology, College of Public Health, East Tennessee State University, Johnson City, TN 37614, USA; quinnm@mail.etsu.edu; 11 Department of Biostatistics and Epidemiology, College of Public Health, Center for Rural Health and Research, East Tennessee State University, Johnson City, TN 37614, USA; wahlquist@mail.etsu.edu; 12 Hopital Necker-Enfants Malades, CF Center, Inserm U1151, Universite Paris Cite, 75015 Paris, France; isabelle.sermet@aphp.fr

**Keywords:** cystic fibrosis, fat-soluble vitamins, exocrine pancreatic insufficiency, vitamin deficiency, vitamin supplementation, DEKAs Plus Liquid, pediatric nutrition, vitamin absorption, nutritional status

## Abstract

Background: Children with cystic fibrosis (CwCF) are at increased risk of fat-soluble vitamin (FSV) deficiency due to fat malabsorption. In France, children were usually supplemented with multiple individual vitamin preparations but have recently converted to DEKAs Plus Liquid (DPL), an absorption-enhanced, CF-specific, multivitamin formulation that includes vitamins D_3_, E, K_1_, A (beta-carotene and retinol), B, C, zinc, and selenium. We evaluated the impact of this change on FSV levels, treatment satisfaction, and compliance. Methods: Young CwCF (n = 39, mean age 2.3 ± 1.1 years) were analyzed retrospectively. Serum FSV levels, prothrombin time, treatment satisfaction, and compliance were assessed at baseline (M0) and after 12 months (M12). Results: Paired data analysis was performed on the 34 who completed 12 months. After 3 months, the initial daily dose was adjusted in nine cases. Median (interquartile range) serum levels of vitamin E (19.3 (17–27) vs. 26.3 (20–31) µmol/L, *p* = 0.0002) and 25-hydroxyvitamin D (80.2 (68–91) vs. 88.5 (79–108) nmol/L, *p* = 0.04) increased. Prothrombin time and vitamin A levels showed no significant change (*p* = 0.7, *p* = 0.4, respectively). The total number of FSV deficiencies decreased from 10 to 1, vitamin D deficiency was eliminated, and vitamin K_1_ supplementation increased from 76% to 100% (*p* = 0.008). The median treatment satisfaction score (VAS_1–10_) improved from 7 (5–9) to 9 (9–10) (*p* = 0.0006). Patient compliance remained high (9.5 (8.5–10) vs. 10 (9–10), *p* = 0.4). Conclusions: Switching from individual vitamins to DPL reduced vitamin deficiencies and improved FSV levels and patient satisfaction.

## 1. Introduction

Children with cystic fibrosis (CwCF) and exocrine pancreatic insufficiency face an increased risk of fat-soluble vitamin (FSV) deficiencies (D, E, K, and A) due to residual fat malabsorption despite pancreatic enzyme supplementation [1]. CF guidelines recommend routine FSV supplementation at age-appropriate daily doses [1]. However, these daily doses often require high-strength vitamin preparations that are simply not available, only available in an unpractically low strength, or not available in a child-friendly pharmaceutical form, e.g., such as a liquid with optimized palatability. For instance, in France, vitamin K_1_ was administered orally from an ampoule intended for intravenous use that is known for its poor taste. Over-supplementation is also a concern. One study found elevated serum retinol (vitamin A) in 58% of children and young adults with CF, with 73% exceeding guideline dosing recommendations [2].

The use of 2–4 individual vitamin preparations increases treatment burden, potentially reducing adherence and treatment satisfaction, though this has not been well studied, especially in the first years of life. Additionally, published vitamin studies in CF often fail to mention the exact products and/or daily doses that are taken by patients, making it difficult to interpret any changes in serum vitamin levels that are reported [2,3,4,5,6,7].

Recently, in France, CwCF transitioned to DEKAs Plus Liquid (DPL), a clinically studied [8,9,10,11,12], absorption-enhanced [13] liquid formulation containing vitamins D_3_, E, K_1_, A (mostly as beta-carotene, which is also known as pro-vitamin A), B, C, zinc, and selenium. FSV daily doses with this formulation align with the latest CF dietary guidelines [1]. The use of beta-carotene reduces the risk of supraphysiological vitamin A levels [14]. Vitamin A at high daily doses is hepatotoxic [15].

We evaluated FSV daily doses and serum levels, patient satisfaction, and patient compliance in CF centers in France in young CwCF who switched from individual vitamin preparations to DPL.

## 2. Materials and Methods

Data from CwCF under four years old at baseline and supplemented with FSV were included in the analysis. There were no further inclusion or exclusion criteria. The CwCF were followed at six centers in France between May 2022 and July 2023 and were retrospectively reviewed for routine vitamin measurement and Visual Analogue Scale (VAS_1–10_) scores at baseline (M0) and 12 months (M12) after switching from 2–4 individual vitamin preparations to DPL (DEKAs Plus Liquid, Callion Pharma, Jonesborough, TN, USA) to assess possible improvements in CF care. For VAS, parents or caregivers were asked to rate two questions from 1 to 10: “How satisfied are you with your child’s current vitamin regimen?” and “Can you assess compliance with the current regimen?” Centers provided anonymized data from 6–7 consecutive patients with parental information for de-identified data sharing, resulting in 39 enrolled patients. For the composition of DPL, see Table 1. The products available orally for young children in France at baseline were diverse and not specific to CF: for vitamin A, mainly preformed vitamin A; for vitamin E, alpha-tocopherol or tocofersolan; for vitamin D, partially D_2_ and/or D_3_; and for vitamin K_1_, IV ampoules.

Vitamin serum levels measured after 3 months were used by clinicians to evaluate if the dosing needed adaptation. All data such as age, length or height, weight, pancreatic enzyme use, fecal elastase-1, vitamin daily doses, serum levels, and VAS_1–10_ scores were obtained from medical records. The following values at both M0 and M12 were extrapolated and used for analysis: FSV supplementation as median daily doses (IU or mg), serum levels of vitamin A (µmol/L), D (nmol/L) and E (µmol/L), prothrombin time expressed in % (PT%) as a surrogate marker of vitamin K status, patient experience as self-reported compliance, and treatment satisfaction reported by the parents (VAS_1–10_). Adjustments of daily doses, treatment discontinuations, and adverse events were assessed qualitatively. Median FSV dosages were compared to those recommended in European CF nutritional guidelines [1]. For infants, the recommended daily doses are 400–1000 IU of vitamin D_3_, 50 IU of vitamin E, and 0.3–1.0 mg of vitamin K_1_. For children > 1 year of age, the recommended daily doses are 800–2000 IU of vitamin D_3_, 100–400 IU of vitamin E, and 1–10 mg of vitamin K_1_. There is no specific daily dose recommendation for vitamin A. According to the guidelines the daily dose must be adapted rapidly to reach the normal serum reference range [1], which happens by itself via a feedback loop, if enough vitamin A is given as beta-carotene.

### Statistical Analysis

Descriptive statistics such as means, standard deviations (SD), medians, interquartile ranges (IQR), frequency counts, and percentages are reported for patient characteristics and laboratory values, as appropriate. Data are expressed in SI units. Wilcoxon signed-rank tests (two-sided) were used to assess changes from M0 to M12 in paired daily vitamin doses and serum levels. A two-sided α = 0.05 level of significance was used for all testing. As Wilcoxon signed-rank tests utilize paired data, the analyses only included cases with available paired data from both M0 and M12. All analyses and results were generated using SAS Software, Version 9.4, of the SAS System for Windows. Deficiencies are presented as the number of serum samples with vitamin A, D, or E deficiency or insufficiency or below-normal PT%. Vitamin deficiencies were defined as follows: retinol < 0.71 µmol/L and α-tocopherol ≤ 11.6 µmol/L, as previously published for young CwCF by [16] and vitamin D deficiency 25OHD < 50 nmol/L, insufficiency 50–74 nmol/L, and sufficiency ≥ 75 nmol/L, according to European CF nutritional guidelines [1]. All centers used a cutoff point for PT% of <70%. Supraphysiological vitamin levels were not analyzed due to a lack of consistent age-adjusted reference values in literature.

## 3. Results

Thirty-nine (39) CwCF followed at six CF centers in France, switched from an individual vitamin regimen to DPL from an individual vitamin regimen. During follow-up assessments, DPL was discontinued before M12 in four cases due to regurgitation (n = 1), agitation (n = 1), or taste issues (n = 2). One additional child switched to the solid formulation, leaving 34 patients to be included in this analysis. Patient demographics and baseline characteristics are shown in Table 2. All were diagnosed with CF via newborn screening, and all but one had exocrine pancreatic insufficiency (fecal elastase-1 < 100 μg/g). All had previously used 2–4 individual fat-soluble vitamin (FSV) supplements. One child, who had paused supplementation due to high vitamin levels a few months before baseline, started DPL after baseline assessment. Two children began lumacaftor/ivacaftor while on DPL, without daily dose adjustments.

Table 3 shows the median vitamin A, D_2_/D_3_, E, and K_1_ daily doses at baseline (M0) and M12, highlighting differences in the dosing regimens. Vitamin D_2_ was predominantly used in the individual vitamin preparations, while vitamin D_3_ was used in DPL. Although the median vitamin D_3_ daily dose was significantly lower at M12 than vitamin D_2_ at M0 (*p* = 0.0005), it remained within the guideline recommendations [1]. Median vitamin E and K_1_ daily doses increased significantly to guideline-recommended levels (*p* = 0.0034, *p* = 0.004, respectively). The inclusion of vitamin K_1_ in DPL increased guideline compliance from 76% to 100% of CwCF (*p* = 0.008). While the preformed vitamin A (retinol) daily dose decreased from M0 to M12, total vitamin A intake almost doubled due to DPL providing a higher daily dose of pro-vitamin A (beta-carotene), a precursor of vitamin A that is converted to vitamin A as needed and does therefore not result in supraphysiological vitamin A levels [14]. Pancreatic enzyme replacement therapy (PERT) increased slightly from 8003 ± 2723 IU/kg/day at M0 to 8406 ± 3481 IU/kg/day at M12 (*p* = 0.97).

At 3 months, DPL dosing was adjusted in nine cases (five increased, four decreased). However, the median daily dose remained stable over 12 months: 2.0 (1.5–2.5) vs. 2.0 (1.2–2.5) mL (*p* = 0.4).

**Figure 1:**
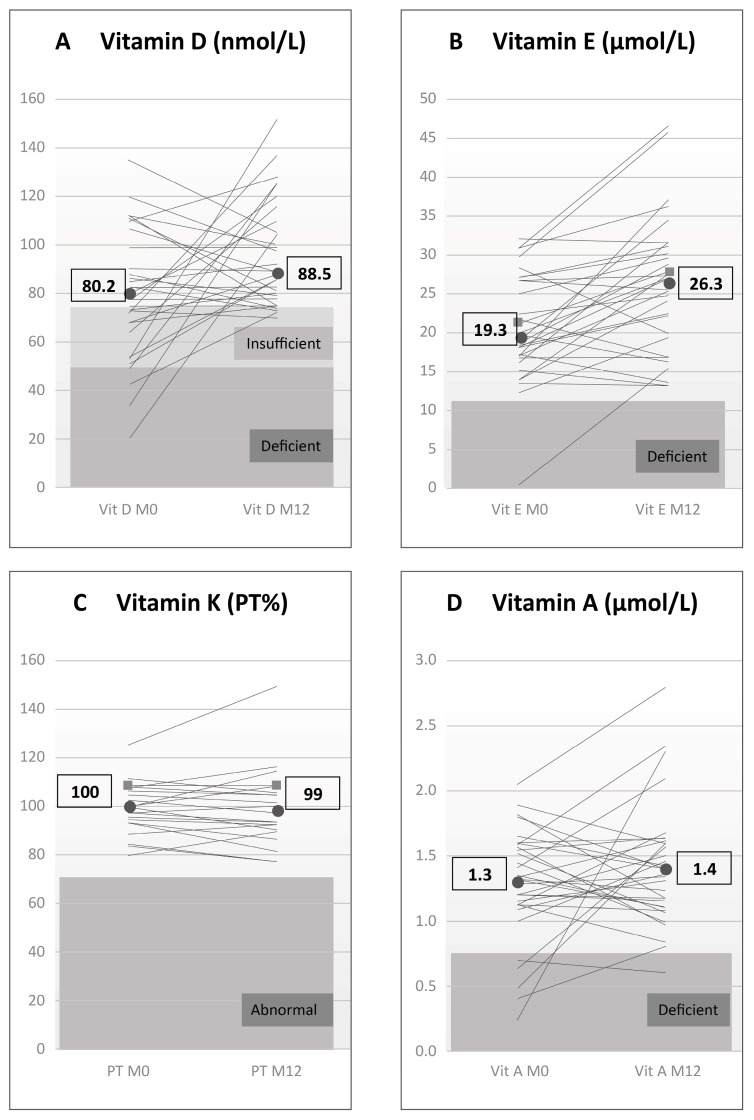
Serum levels of vitamins D (panel (**A**)), E (panel (**B**)), and A (panel (**D**)) and a surrogate parameter for vitamin K levels—prothrombin time expressed in % (PT%) (panel (**C**)) at baseline and 12 months after switch to DPL (DEKAs Plus Liquid). Grey panels mark areas of deficiency. The light grey panel marks the area of insufficiency. Boxed values represent median values. See text for significances.

Figure 1A–D presents the median serum levels of vitamins D, E, and A at M0 and M12, along with prothrombin time (PT%) as an indirect measure of vitamin K sufficiency. 25-Hydroxyvitamin D levels increased significantly (80.2 (68–91) → 88.5 (79–108) nmol/L, n = 32, *p* = 0.04). Vitamin E levels increased significantly (19.3 (17–27) → 26.3 (20–31) µmol/L, n = 32, *p* = 0.0002). Vitamin A levels did not change (1.3 (1.1–1.6) vs. 1.4 (1.1–1.6) µmol/L, n = 32, *p* = 0.4). At M12, PT% also remained unchanged (100 (95–105) vs. 99 (91–106) %, n = 23, *p* = 0.7).

Table 4 shows that the number of deficiencies in any FSV decreased from 10 (M0) to 1 (M12). By M12, vitamin D deficiency was eliminated, with all four previously deficient CwCF improving. One patient remained deficient in vitamin A (see Figure 1D). Table 4 also shows that the number of insufficiencies or deficiencies in any FSV decreased from 19 (M0) to 5 (M12). Of these five remaining insufficiencies or deficiencies, four were borderline insufficiencies in vitamin D, with serum values ranging from 70–74 nmol/L, all very close to cut-off point of 75 nmol/L for vitamin D sufficiency (see Figure 1A).

Treatment satisfaction improved significantly (VAS_1–10_ 7 (5–9) → 9 (8.5–10), n = 24, *p* = 0.0006), despite minor concerns about taste (n = 4) and the dosing pipette (n = 3). Compliance was also high throughout this study (VAS_1–10_ 9.5 (8.5–10) → 10 (9–10), n = 24, *p* = 0.4).

## 4. Discussion

These data show, for the first time, that switching young CwCF from multiple individual vitamin preparations to a CF-specific formulation improves FSV status and increases patient satisfaction. The CF-specific formulation aligned the median FSV dosing with CF nutritional guidelines for vitamins E and K and ensured that all patients received the preferred vitamin D_3_ instead of D_2_. The number of patients receiving vitamin K supplementation increased to 100%. Serum levels of vitamins D and E increased significantly. Median serum vitamin A levels remained stable, but four out of five deficiencies were resolved. The total number of FSV deficiencies decreased, and vitamin D deficiency was eliminated.

With DPL, vitamin E dosing increased significantly to guideline levels, serum levels rose accordingly, and the one previously deficient CwCF achieved sufficiency. DPL contains a special form of vitamin E that is well-absorbed in CF [8] and in bile-deficient conditions like cholestasis [17,18], where it enhances the uptake of vitamin D [18,19] and A [18,20], and is widely used as a solubilizer and absorption enhancer for fat-soluble drugs [13]. This might explain why despite a 25% reduction in the median daily dose of vitamin D using DPL, serum levels increased significantly, and vitamin D deficiency was eliminated. However, this finding could also be explained by the switch from, mostly, the less potent vitamin D_2_ [21] to vitamin D_3_ or a combination of both. An increase in compliance seems less likely as an explanation, as this was reported to be consistently high in both regimens.

With DPL, vitamin K dosing increased significantly to guideline levels [1], partly because of an increased daily dose and partly because the percentage of patients taking vitamin K increased to 100%. This last factor was most likely due to the better palatability of DPL. Prothrombin time was not influenced by any of this, most likely because it is not a very sensitive parameter for vitamin K status, unlike protein induced by vitamin K absence or antagonist-II (PIVKA-II) or undercarboxylated (i.e., inactive) osteocalcin levels, which are not routinely performed.

While the preformed vitamin A (retinol) daily dose decreased by 75% with DPL, the total vitamin A intake almost doubled due to a higher daily dose of beta-carotene. This is a precursor of vitamin A, also known as pro-vitamin A, that is converted to vitamin A as needed, mainly by enterocytes of the small intestine [14], and has proven to be safe and effective in CwCF [22]. This likely explains why four out five deficient patients became sufficient, while the median serum retinol levels remained stable. This regulatory function of beta-carotene is a relevant safety feature that prevents supraphysiological levels of vitamin A, which are potentially hepatotoxic and unsafe during pregnancy and have been reported in CwCF [2,23].

We can only speculate about the reasons why patients preferred taking a once-daily CF specific multivitamin over taking 2–4 individual FSV supplements, some of which had to be taken daily and others at other intervals. Several reasons seem plausible: simplicity, ease of use, and palatability. Palatability was not optimal for all patients as two CwCF discontinued treatment for taste and four CwCF mentioned that the taste was not optimal but continued treatment nonetheless. Caregivers should be advised that some of this might be remedied because DPL, being water-soluble, can be mixed with any liquid, be it (luke) warm or cold. Providing a taste for long-term daily use that is liked by everyone remains a challenge.

Although the median daily dose of DPL remained unchanged throughout the study period, the data illustrate that monitoring FSV serum levels within 3 months after initial dosing is important because an adjustment of the daily dose was made in 26,5% of cases, with almost equal numbers requiring an increase or decrease in daily dose.

Comparisons of these results with other studies are challenging, as we know of no published research specifically examining the impact of switching from individual vitamin preparations to a CF-specific multivitamin in young CwCF. Additionally, most CF vitamin studies [2,3,4,5,6,7] lack the detailed documentation on supplement composition and dosing that this study provides, probably based on the assumption that differences in formulation among vitamin supplements have no impact on serum levels.

This study has several limitations. First, the sample size is relatively small but consistent with nutritional studies in CwCF [2,3,11,22], in which very young children are usually underrepresented in the cohorts. The sample size of these references ranges from 24 [22] to 78 [2] participants. Second, changes in dietary vitamin intake or additional supplements were not considered. It is unlikely that these might have influenced the outcomes, as young CwCF are likely to take standardized dietary food regimens, and there is little reason to believe that these regimens changed after the switch to DPL. In addition, considering the high daily doses of vitamins that are needed in CwCF, it is unlikely that a change in diet could have increased vitamin serum levels in a meaningful way. As for possible additional supplementation, in France, all supplements needed by CwCF are fully reimbursed, making it unlikely that parents of CwCF will give their child unprescribed vitamin supplements for which they have to pay out of pocket. This is a contextual limitation; practices may differ in other countries with different reimbursement models.

Finally, since mid-2024, about half of the participants in this study would currently be using elexacaftor/tezacaftor/ivacaftor (ETI) therapy, and this limits the generalizability of these results to current daily practice. However, the putative impact of ETI on vitamin needs in CF, if any, are uncertain, as the data are conflicting [3,5,7,24].

In conclusion, switching young CwCF from multiple individual vitamin preparations to a CF-specific formulation improves FSV status and increases patient satisfaction, resulting in a clinically meaningful improvement in the quality of nutritional care. Additional studies into the role of CF-specific vitamin formulations in CwCF are warranted to confirm these findings.

## Figures and Tables

**Table 1:** Composition of DPL (DEKAs Plus Liquid).

Ingredient	Unit	Amount per 1 mL
Vitamin A as retinyl palmitate	IU (μg RE) *	750 (225)
Vitamin A as ß-carotene	IU (mg RE) *	5001 (1.5)
Vitamin D_3_	IU (μg)	750 (18.8)
Vitamin E	IU (mg)	50 (33.6)
Vitamin K_1_	μg	500
Thiamin (B_1_)	mg	0.6
Riboflavin (B_2_)	mg	0.6
Niacin	mg	6
Vitamin B_6_	mg	0.6
Vitamin C	mg	45
Biotin	μg	15
Pantothenic Acid	mg	3
Zinc	mg	5
Selenium	μg	10

* RE = all trans retinol equivalent.

**Table 2:** Patient demographics and baseline characteristics (n = 34).

Characteristic	
Age (years) *	2.0 ± 1.1
Female	13/34 (38%)
Length or height (cm) *	84.1 ± 11.8
Weight (kg) *	11.0 ± 3.1
Pancreatic Insufficiency (n) (%) **	33/34 (97%)
Pancreatic enzyme replacement therapy (IU/kg/day) *	8003 ± 2723

* Mean ± SD ** Defined as fecal elastase-1 < 100 μg/g).

**Table 3:** Median (IQR) fat-soluble vitamin dose per day (n = 34).

Daily Dose	Pairs (n)	Baseline (M0)	Month 12 (M12)	*p-Value*
Vitamin D (IU) ^1^	34	D_2_ 2000 (2000–3000)	D_3_ 1500 (900–1875)	0.0005
Vitamin E (IU) ^1^	34	16 (16–92)	100 (60–125)	0.0034
Vitamin K_1_ (mg) ^1^	34	0.3 (0.1–1.4)	1.0 (0.6–1.3)	0.004
Vitamin A, total (IU) ^1^	34	6000 (4500–6000)	11,502 (6901–14,378)	<0.0001
Vitamin A as retinol (IU)	34	6000 (4500–6000)	1500 (900–1875)	<0.0001
Vitamin A as β-carotene (IU)	34	0 ^2^	10,002 (6001–12,503)	<0.0001
DPL * (mL)	34	0 ^2^	2.0 (1.2–2.5)	

* DEKAs Plus Liquid ^1^ Recommended daily doses in ECFS guidelines are given in the Materials and Methods section. ^2^ Not provided in the regimen at baseline.

**Table 4:** Number of samples with deficient, insufficient, or sufficient values of vitamins D, E, or A or a normal or abnormal prothrombin time (%) at baseline and at month 12.

		Cutoff Value	Baseline (n)	Month 12 (n)
Vitamin D	Deficient	<50 nmol/L	4	0
	Insufficient	50–74 nmol/L	9	4
	Sufficient	≥75 nmol/L	21	28
Vitamin E	Deficient	<11.6 µmol/L	1	0
	Sufficient	≥11.6 µmol/L	33	32
Prothrombin time	Abnormal	<70%	0	0
	Normal	≥70%	28	28
Vitamin A	Deficient	<0.71 µmol/L	5	1
	Sufficient	≥0.71 µmol/L	29	31

## Data Availability

The data presented are unavailable in their original forms as they originate from patient records stored in the participating centers. Most data are included in the article material. Further inquiries can be directed to the corresponding author(s).

## Abbreviations

The following abbreviations are used in this manuscript:

CF	Cystic fibrosis
CwCF	Children with cystic fibrosis
DPL	DEKAs Plus Liquid
ECFS	European Cystic Fibrosis Society
F	Female
FSV	Fat-soluble vitamins
IQR	Interquartile range
M	Male
M0	Baseline, month 0
M12	Month 12 after baseline
PERT	Pancreatic enzyme replacement therapy
VAS_1–10_	Visual Analogie Scale score (range 0–10 points)
